# Effects of Training for Finger Perception on Functional Recovery of Hemiplegic Upper Limbs in Acute Stroke Patients

**DOI:** 10.1155/2019/6508261

**Published:** 2019-11-04

**Authors:** Naho Umeki, Jun Murata, Misako Higashijima

**Affiliations:** Department of Physical and Occupational Therapy, Graduate School of Biomedical Sciences, Nagasaki University, 1-7-1 Sakamoto, Nagasaki 852-8520, Japan

## Abstract

**Background:**

Stroke causes severe disability, including motor and sensory impairments. We hypothesized that upper limb functional recovery after stroke may be augmented by combining treatments for motor and sensory functions. In order to examine this hypothesis, we conducted a controlled trial on rehabilitation for sensory function to the plegic hand.

**Methods:**

The sensory training program consisted of several types of discrimination tasks performed under blind conditions. The sensory training program was performed for 20 min per day, 5 days a week. An experimental group of 31 patients followed this sensory program, while a control group of 25 patients underwent standard rehabilitation. The efficacy of the intervention was evaluated by the tactile-pressure threshold, handgrip strength, and the completion time of manipulating objects. A two-way repeated measures analysis of variance was used to assess interactions between group and time. Moreover, to provide a meaningful analysis for comparisons, effect sizes were calculated using Cohen's *d*.

**Results:**

The mean change in the tactile pressure threshold was significantly larger in the experimental group than in the control group (*p* < 0.05, *d* = 0.59). Moreover, the completion times to manipulate a middle-sized ball (*d* = 0.53) and small ball (*d* = 0.80) and a small metal disc (*d* = 0.81) in the experimental group were significantly different from those in the control group (*p* < 0.05).

**Conclusion:**

The present results suggest that the sensory training program to enhance finger discrimination ability contributes to improvements in not only sensory function but also manual function in stroke patients. The trial is registered with the UMIN Clinical Trials Registry (UMIN000032025).

## 1. 1. Introduction

Hand function, which is excellent at performing motor and sensory functions, is affected by neurological disturbances (e.g., disorders of the central nervous system and peripheral nerves). Stroke mostly occurs in the elderly and causes severe disability, including motor and sensory impairments, such as muscle weakness, decreased range of motion [1–3], and an inability to discriminate tactile and proprioceptive sensations [4–6].

The goals of rehabilitation after stroke are to improve function, thereby allowing stroke patients to become as independent as possible. A stroke rehabilitation program focuses on the relearning of basic skills that may have become impaired, such as bathing, eating, dressing, and walking. Motor deficits are the primary reason for functional disability. Therefore, therapeutic approaches based on motor learning paradigms have been employed to facilitate the recovery of impaired movement in patients [7–10]. Approximately 50% of stroke patients have hand sensory impairments, particularly in tactile and proprioceptive discrimination [4, 6]. However, previous studies reported that sensory impairments have not yet been examined in sufficient detail because of the emphasis placed on the motor outcomes of stroke patients [11]. We recently demonstrated that the severity of sensory impairments in stroke patients was related to the recovery process of motor impairments [12]. Functional recovery after stroke may be augmented by combining treatments for motor and sensory functions. Therefore, the aim of the present study was to clarify whether the recovery of upper limb function is enhanced by additionally performing training aimed at improving sensory function.

## 2. Materials and Methods

### 2.1. Participants

Subjects were hospitalized and receiving therapeutic interventions at the rehabilitation unit. The following inclusion criteria were used: (1) first time stroke; (2) a Revised Hasegawa Dementia Scale (HDS-R) [13] score higher than 21 (mean score: 28.2±2.2); (3) no severe cognitive deficits that preclude clinical evaluations, such as aphasia and unilateral neglect; and (4) no other serious medical conditions.

A quasirandomized 2-group pretest-posttest was used to examine the effects of the sensory training program in addition to standard rehabilitation therapy on paralyzed upper limbs. During the research period between July 2015 and January 2016, thirty-one subjects participated in the experimental group that performed the sensory training program in addition to standard rehabilitation therapy. After the end of this research period, an additional 25 patients were enrolled during the research period between February 2016 and August 2017 as the control group that received standard rehabilitation therapy without the sensory training program. Their demographic data are shown in Table 1.

Experimental procedures were explained to all subjects in advance, and written consent was obtained. The present study was performed in accordance with the Declaration of Helsinki and approved by the Institutional Ethical Committees of Nagasaki University and Nagasaki Medical Center.

### 2.2. Common Intervention

All patients participated in a standard rehabilitation program, which consisted of 40 min of physical therapy and/or occupational therapy per day, 6 days a week. Physical therapy focused on gait and exercise related to the activities of daily living. This program included passive range of motion exercise for the affected side and muscle strength exercise for the unaffected side. Occupational therapy included upper limb exercise and self-care skill training to maximize the ability to perform the activities of daily living.

### 2.3. Group-Specific Intervention

The experimental group performed the sensory training program in addition to the aforementioned program (standard rehabilitation program). The sensory training program consisted of two types of discrimination tasks as follows: (1) touch discrimination task to identify different surfaces (sandpaper: No. 80, No. 120, and No. 320) and materials (cloth: fur, satin fabric, and linen cloth) and (2) a braille-dot counting task in which subjects counted the number of dots in a series of random Braille letters (subjects were not asked to read the letters). In each task, subjects were presented with a stimulus set under visual control for 1 min, and then one of the set was passively presented for 15 sec under blind conditions. The subject was then asked to provide an answer to the task and was given oral feedback by a trainer as to whether the answer was correct for each set of surfaces. The sensory training program was performed by the experimental group for 20 min per day, 5 days a week.

### 2.4. Outcome Measurements

We assessed motor recovery of the hand using Brunnstrom's 6 stages judged by clinical observations [14, 15]. Brunnstrom's stages describe a commonly observed sequence of motor recovery after stroke based on the degree of spasticity and the appearance of voluntary movement. Higher stages indicate better recovery.

Handgrip strengths were measured on both sides (impaired and unimpaired sides) using a handgrip dynamometer (TKK5401; Takei Kiki Kogyo, Japan). These tests were repeated twice, and the maximum value was recorded.

The tactile pressure threshold on both sides at the distal palmer pad of the index finger was evaluated using Semmes-Weinstein monofilaments (North Coast Medical, Inc., Morgan Hill, CA, USA). This measurement was performed on both hands (impaired and unimpaired sides). We used 20 types of filaments ranging in weight from 0.004 to 447 g. The aesthesiometer pressure (g) of each filament was converted to log_10_0.1 mg, yielding a scale composed of intervals of approximately equal intensities between filaments. Subjects were tested with their eyes closed after receiving clear instructions. The target area was marked on the volar side of the distal phalanx of the index finger. Each filament was pushed into the target area until it bent by approximately 90° for approximately 1 second. The threshold was recorded as the smallest filament diameter that was perceived in at least 80% of its applications (5 trials).

We measured the completion time of manipulating objects using one upper limb (impaired side) as an index of manual function. Subjects were instructed to grasp or pinch objects of 4 different shapes and sizes and to carry them to a designated area. The objects were large balls (70 mm in diameter, *n* = 5), middle-sized balls (40 mm in diameter, *n* = 6), small balls (5 mm in diameter, *n* = 6), and small metal discs (20 mm in diameter × 2 mm in height, *n* = 6). The distances to carry the objects were 50 cm for the large and middle-sized balls and 30 cm for the small balls and small metal discs. The time to complete the task was evaluated with an upper limit of 30 seconds.

### 2.5. Statistical Analysis

Differences in handgrip strength and the tactile pressure threshold due to hemiparesis after stroke were examined using the paired *t*-test. Moreover, a two-way repeated measures analysis of variance was used to assess interactions between group and time. If an interaction was present, the unpaired *t*-test was used to compare changes in measurement data from pre- to postintervention between the groups. The level of significance was defined as *p* < 0.05. Data were expressed as the mean ± standard deviation. To provide a meaningful analysis for comparisons, effect sizes were calculated using Cohen's *d* [16, 17]. The strength of the effect size was assessed as small (<0.50), moderate (0.50-0.79), or large (>0.80).

## 3. Results

### 3.1. Differences in Handgrip Strength and the Tactile Pressure Threshold between Impaired and Unimpaired Sides after Stroke

Table 1 shows the frequency of Brunnstrom's recovery stages. Most patients were in the higher recovery stages (5 or 6). The handgrip strengths of the impaired and unimpaired sides were 18.4 ± 12.1 and 26.4 ± 9.6 kg, respectively (*n* = 56). The unpaired *t*-test revealed a significant difference in handgrip strength between the impaired and unimpaired sides (*p* < 0.01). The tactile pressure threshold was significantly higher on the impaired side (3.58 ± 0.82 log_10_0.1 mg) than on the unimpaired side (3.08 ± 0.47 log_10_0.1 mg) (*p* < 0.01).

### 3.2. Between-Group Comparisons

The mean values for each variable in the control and experimental groups pre- and postintervention are summarized in Table 2. No significant differences were observed in any measurement data before the intervention. After the intervention, the two-way ANOVA showed a significant interaction for the tactile pressure threshold and the completion time of manipulating middle-sized and small balls and small metal discs but not for handgrip strength or the completion time of manipulating large balls. The mean change in the tactile pressure threshold was larger in the experimental group (−0.32 ± 0.36 log_10_0.1 mg) than in the control group (−0.12 ± 0.29 log_10_0.1 mg) (*p* < 0.05). Moreover, the completion times of manipulating middle-sized and small balls and small metal discs in the experimental group(−2.0 ± 4.3, −4.4 ± 5.3, and −3.5 ± 4.1 sec, respectively) were significantly different from those in the control group (0.3 ± 3.9, −1.0 ± 2.5, and −0.4 ± 2.8 sec, respectively) (*p* < 0.05).  Table 3 shows the effect size for each dependent measure across groups. A large intervention effect was observed for the completion time of manipulating small balls (*d* = 0.80) and small metal discs (*d* = 0.81), while a moderate intervention effect was observed for the tactile pressure threshold (*d* = 0.59) and the completion time of manipulating middle-sized balls (*d* = 0.53). On the other hand, the effect size for handgrip strength (*d* = 0.35) and the completion time of manipulating large balls (*d* = 0.29) showed a small intervention effect.

## 4. Discussion

The aim of the present study was to clarify whether functional recovery of the upper limbs is enhanced by combining treatments for motor and sensory functions in acute stroke patients. The main results obtained were the greater reductions observed in the tactile pressure threshold and completion time of manipulating objects in the experimental group. These results suggested that the sensory training program to enhance finger discrimination ability contributed to improving not only sensory function but also manual function in stroke patients.

Subjects with hemiplegia after stroke have impaired motor activity and muscle tone [2, 3], muscle weakness [1], and abnormal sensations [4–6]. Significant decreases in muscular strength and tactile sensitivity were observed on the impaired side in the present study. A previous study [18] reported that the mean values of handgrip strength and the tactile-pressure threshold in age-matched (65-69 yr) healthy females were 22.1 ± 3.8 kg and 2.8 ± 0.3 log_10_0.1 mg, respectively. Using these data as a control, only a slight difference was noted from measurements on the unimpaired side (26.4 ± 9.6 kg and 3.0 ± 0.5 log_10_0.1 mg, respectively). Since this study included male subjects, grip strength on the unimpaired side was slightly higher. However, marked differences were observed from handgrip strength and the tactile-pressure threshold on the impaired side (18.4 ± 12.1 kg and 3.6 ± 0.8 log_10_0.1 mg, respectively). These functional impairments have been implicated in the poor performance of the activities of daily life [19–21]. Therefore, a long-term continuous rehabilitation approach combining physical and occupational therapies is often needed by these patients.

Early rehabilitation is widely regarded as an important feature of effective stroke care. Thus, recommendations that rehabilitation begin as soon as possible are common in clinical guidelines [22–24]. The present results revealed that Brunnstrom's stages, which are used to assess motor recovery in stroke, were improved after acute stroke rehabilitation (change in the average value from 5.1 ± 1.6 to 5.4 ± 1.2, *n* = 56, *p* < 0.05). Previous studies reported that early rehabilitation is independently associated with good functional outcomes at 3 and 12 months [25, 26]. These findings suggest that early rehabilitation for acute stroke patients promotes functional motor recovery.

The effects of early rehabilitation have been noted not only in motor function but also in sensory function. The present results indicated that the tactile-pressure threshold of the hand decreased after the rehabilitation period. A decrease in this threshold reflects improvements in sensory sensitivity. Furthermore, this effect on the tactile-pressure threshold was more prominent in the experimental group (decreased by −0.32 ± 0.36 log100.1 mg) than in the control group (decreased by −0.12 ± 0.29 log100.1 mg). Regarding the recovery of sensory impairments after stroke, previous studies reported that somatosensory deficits were improved by training using tactile, proprioceptive, and object recognition tasks [5, 11, 27]. These findings suggest that specific training to facilitate sensations in the plegic hand effectively improves somatosensory deficits.

The decreases observed in the manipulation times of small balls and small metal discs were markedly greater in the experimental group than in the control group. These results suggest that improvements in somatosensory deficits after stroke reflect the control of dexterous finger movements. Sensory feedback information is an important element for motor control. Previous studies reported that the absence of cortical activation by a sensory stimulation was associated with poorer outcomes in stroke patients and that cortical activity in response to somatosensory input predicted late motor outcomes in the early poststroke phase [28, 29]. Moreover, we previously demonstrated that the deterioration of manual function in elderly patients was closely associated with a decline in tactile sensibility rather than a change in muscular strength in the hand [18]. The control of manual function, which requires accuracy, is affected by the amount of sensory feedback information arising from skin sensory receptors.

The present results need to be interpreted carefully due to some limitations. We only investigated the sensitivity of light touch sensations. Sensory impairments following stroke include the loss of not only tactile sensations but also protective and proprioceptive sensations [5, 6]. Therefore, the effects of specific training to facilitate sensations for sensory impairments other than tactile sensations currently remain unclear. Moreover, the types and degrees of disabilities that develop after a stroke depend on which area of the brain is damaged. In the present study, the severity of motor and sensory impairments differed in each subject. However, the sample size of the present study was too small to reveal the influence of the types and degrees of disabilities on sensory training. These preliminary results need to be replicated with larger samples.

## 5. Conclusions

In conclusion, recovery of manual function in stroke patients is affected by improvements in sensory function. Training aimed at improving not only motor function but also sensory function needs to be considered in stroke rehabilitation.

## Figures and Tables

**Table 1:** Demographic characteristics of subjects (*N* = 56).

	Experimental group (*N* = 31)	Control group (*N* = 25)
Age (years)	65.2 ± 12.2	65.3 ± 14.1
Gender (male/female) (number)	19/12	14/11
Time post-stroke (days)	5.6 ± 4.0	6.4 ± 6.8
Rehabilitation training period (days)	15.5 ± 6.3	16.3 ± 8.1
Hemiparetic side (right/left) (number)	17/14	14/11
Type of stroke (hemorrhage/infarction) (number)	21/10	11/14
Brunnstrom's stage (number)	I (3), II (0), III (1),IV (2), V (4), VI (21)	I (2), II (1), III (2),IV (1), V (3), VI (16)
Lesion site (number)	BG (7), BS (4), IC (5), SAH (4), TL (3), ICA (2), MCI (3), MCA (2), PCA (1)	BG (10), BS (1), IC (4), SAH (3), TL (1), MCI (3), MCA (2), PG (1)

BG: basal ganglia; BS: brainstem; IC: internal capsule; SAH: subarachnoid hemorrhage; TL: thalamus; ICA: internal carotid artery; MCI: multiple cerebral infarctions; MCA: middle cerebral artery; PCA: posterior cerebral artery; PG: precentral gyrus. Values are shown as means ± standard deviations.

**Table 2:** Changes observed in parameters measured.

	Experimental group (*N* = 31)		Control group (*N* = 25)	
	Pre	Post	Pre	Post
Handgrip strength (kg)	18.9 ± 10.7	21.9 ± 10.7	17.7 ± 13.9	19.4 ± 14.6
Tactile-pressure threshold (log_10_0.1 mg)^∗^	3.51 ± 0.62	3.19 ± 0.51	3.66 ± 1.02	3.54 ± 1.11
Completion time (sec)				
Large ball	12.3 ± 9.0	10.3 ± 8.1	13.9 ± 10.4	12.9 ± 10.6
Middle-sized ball^∗^	11.5 ± 9.0	9.5 ± 8.3	13.1 ± 10.9	13.4 ± 11.7
Small ball^∗^	22.0 ± 7.7	17.6 ± 7.3	20.8 ± 8.2	19.8 ± 8.6
Small metal disc^∗^	21.5 ± 9.5	18.0 ± 8.3	19.7 ± 8.1	19.3 ± 9.0

Values are shown as means ± standard deviations. ^∗^Significant group × time interaction.

**Table 3:** Effect size and clinical significance.

Variable	Effect size	Intervention effect
Handgrip strength (kg)	0.35	Small
Tactile-pressure threshold (log_10_0.1 mg)	0.59	Moderate
Large ball (sec)	0.29	Small
Middle-sized ball (sec)	0.53	Moderate
Small ball (sec)	0.80	Large
Small metal disc (sec)	0.81	Large

## Data Availability

The data used to support the findings of this study are restricted by the Ethical Committees of Nagasaki University and Nagasaki Medical Center in order to protect patients. Data are available from the corresponding author for researchers who meet the criteria for access to confidential data.
